# The Influence of the Periodontal Breakdown over the Amount of Orthodontic Force Reaching the Dental Pulp and NVB During Orthodontic Movements—A Biomechanical Finite Element Analysis

**DOI:** 10.3390/jcm14062094

**Published:** 2025-03-19

**Authors:** Radu-Andrei Moga, Cristian Doru Olteanu, Ada Gabriela Delean

**Affiliations:** 1 Department of Cariology, Endodontics and Oral Pathology, School of Dental Medicine, University of Medicine and Pharmacy Iuliu Hatieganu, Str. Motilor 33, 400001 Cluj-Napoca, Romania; ada.delean@umfcluj.ro; 2 Department of Orthodontics, School of Dental Medicine, University of Medicine and Pharmacy Iuliu Hatieganu, Str. Avram Iancu 31, 400083 Cluj-Napoca, Romania

**Keywords:** periodontal breakdown, neuro-vascular bundle, dental pulp, orthodontic movement, stress absorption–dissipation, orthodontic force

## Abstract

**Background/Objectives**: Most orthodontic forces are absorbed–dissipated before reaching the dental pulp and its neuro-vascular bundle (NVB); nonetheless, no data are available about this issue during the periodontal breakdown. The current study’s objective was to investigate how much orthodontic force reaches the dental pulp and NVB during the orthodontic movements in periodontal breakdown. **Methods**: Herein, an assessment was performed on the second lower premolar of nine patients (72 3D models) and included 1440 numerical simulations. A gradual horizontal periodontal breakdown (1–8 mm loss) was simulated. Five orthodontic movements (intrusion, extrusion, rotation, translation, and tipping) under 0.5 N/5 KPa and 4 N/40 KPa were assessed. The numerical methods used were Von Mises/VM (overall homogenous) and Tresca (shear non-homogenous), suitable for the ductile resemblance of dental tissues. **Results**: Both methods showed similar color-coded projections for the two forces. Quantitatively, Tresca was 1.14 times higher than VM and lower than the maximum physiological hydrostatic circulatory pressure. During the bone loss simulation, the NVB stress was 5.7–10.7 times higher than the pulpal stress. A gradual tissue stress increase was seen, strictly correlated with the bone loss level. For 1 mm bone loss, only 2–3% of the applied force manifested at the NVB level (0.27–0.5% for pulp), while for 8 mm loss, the received stress was 4–10% for the NVB (0.6–0.9% for pulp) when compared to the applied force. Only translation displayed pulpal stress. **Conclusions**: When assessing NVB stress, the tooth absorption–dissipation ability of dental tissues varied between 90 and 93% (8 mm loss) and 97% (1 mm bone loss) and 99% when assessing pulpal stress.

## 1. Introduction

Orthodontic movement is triggered by the various-intensity local circulatory disturbances produced in the rich vessel’s dental tissues: neurovascular bundle/NVB, periodontal ligament/PDL, and dental pulp [1,2,3]. Depending on the amount, intensity, and time, the orthodontic loads applied induce various stress levels, triggering movements [3,4,5,6,7].

Periodontal breakdown influences movement, since the bone and PDL play a main support role, due to biomechanical changes in stress distribution. However, data on periodontal breakdown influences are scarce. The same loads can be safely applied in an intact periodontium but can induce ischemic risks in a reduced periodontium. Additionally, some movements are reported to be more stressful than others. Nevertheless, there is no consensus regarding this issue, with contradictory reports signaling intrusion, translation, or rotation to be more stressful for the intact periodontium and no data for periodontal breakdown cases [8,9].

The clinical orthodontic forces used in the intact periodontium are usually of light and moderate intensity [10]. However, there is no consensus on the optimal force to be safely applied in the intact periodontium and no data for the reduced periodontium. Most orthodontic loads are absorbed and dissipated before reaching the NVB, pulp, and PDL [11]. The absorption–dissipation process also involves dentine, enamel, and stainless-steel brackets [8,9]. Thus, as a result, most of the stresses that reach the NVB, PDL, and dental pulp are below the local physiological maximum hydrostatic pressure (16–22 KPa) [3,4,5,6,7]. However, if the MHP (local maximum physiological hydrostatic pressure) is exceeded, the localized areas of ischemia would trigger the local remodeling process [12,13], inducing orthodontic movement [1,2,3,10,14,15]. In the intact periodontium and healthy intact tissues, light and/or moderate applied forces induce limited localized ischemia areas with no further consequences due to the local tissular ability to sustain, limit, and recover [3,16,17,18]. However, if there is previous occlusal trauma (for PDLs and NVBs) [19,20,21] and dental pulp injuries due to direct/indirect dental pulp capping [3,22,23,24,25], this ability to sustain the damage is severely diminished, leading to ischemia, necrosis, and further tissular loss [15,22,25,26,27,28,29,30]. Nevertheless, these types of lesions [15,16,18,31] are rarely clinically visible and most of them pass unnoticed [19,22], with the effect visible only after the irreversible pathological process began [19,22,31]. The periodontal breakdown induces biomechanical changes in the local stress display, leading to different consequences depending on the bone loss levels [8,9,11,32,33,34]. Thus, a safely applied force in the periodontium and healthy intact tissues [12,13] can induce severe ischemia and necrosis in cases of a reduced periodontium and previous local trauma and/or pulpal injuries [15,16,17,18,27,28,29,30,35,36].

To obtain data on the above-mentioned processes, the biomechanical behavior of each component sensitive to ischemia, such as the NVB and pulp, should be individually assessed in various levels of bone loss. This can be achieved only through numerical studies [8,9,32,33,34,37]. To the best of our knowledge, no other studies except our previous works [8,9,11,32,33,34] had a similar approach. Numerical studies use anatomically accurate 3D tissular models, which are extremely versatile regarding the experimental condition, enabling multiple changes in variables and various results [8,9,32,33,34,37], with remarkable accuracy. However, each numerical study is highly dependent on the anatomical accuracy of the models, the suitable numerical method, and boundary assumptions. The numerical method has been widely used in dentistry, but studies have reported results with accuracy issues contradicting the known clinical data [4,5,6,7,15,16,17,18,35,38,39,40,41,42,43,44,45,46,47,48,49,50,51,52,53]. These results were a result of the misunderstanding of numerical methodology and misuse of brittle and hydrostatic [4,5,6,7,15,16,17,18,35,38,39,40,41,42,43,44,45,46,47,48,49,50,51,54] failure criteria for describing the biomechanical behavior of ductile resemblance tissues [8,9,32,33,34], associated with reduced anatomical accuracy of models as well as a lack of correlations with the MHP and clinical data. Despite a few dental numerical studies signaling these issues [8,9,11,32,33,34], most of the newer reports suffer from the same above-mentioned flaws [48,49,50,51,55,56,57,58]. Additionally, there are also studies reporting the methodology-related poor quality of multiple in vivo studies [13,14,35]. Thus, to obtain accurate data, dental numerical studies must follow all the mandatory engineering field requirements, and the results must be indirectly validated by correlations with physiological constants such as the MHP and known clinical data [59]. It must be remembered that orthodontic forces induce a cascade of biological responses in the periodontal ligament and alveolar bone, leading to bone resorption on the compression side and bone deposition on the tension side. This process is mediated by mechanotransduction, where mechanical stress stimulates the release of inflammatory cytokines (e.g., IL-6, TNF-α) and key regulators like the receptor activator of nuclear factor kappa-Β ligand (RANKL), which promotes osteoclast differentiation and bone remodeling [59].

This study aimed to assess how much orthodontic force applied at the bracket levels during the gradual horizontal periodontal breakdown would affect the NVB and dental pulp level during five orthodontic movements.

## 2. Materials and Methods

This research is part of a larger stepwise research project (clinical protocol 158/02.04.2018) aiming to study orthodontic movements and biomechanical behavior during periodontal breakdown [8,9,11,32,33,34].

Here, research focused on assessing how much of the applied force will reach the NVB and pulp in a gradual horizontal periodontal breakdown during orthodontic movements. The study involved the second lower premolars of nine patients (72 3D models), four males/five females (mean age 29.81 ± 1.45), totaling 1440 numerical simulations. The sample size was nine, higher than most dental numerical studies [4,5,6,7,38,39,40,41,42,43,44,45,46,47,48,49,50,51,52,53,54,55,57,58].

The inclusion criteria were a complete mandibular dental arch in the analyzed region (first and second lower premolars and first lower molar), no malposition, intact healthy teeth, up to 1–2 mm of bone loss, a non-inflamed periodontium, orthodontic treatment indication, and proper oral hygiene.

The exclusion criteria were particular root geometry, abnormal crown shape, root surface defects, radiologically visible bone defects, an abnormal pulp chamber and root canals, more than 2 mm of bone loss, and poor oral hygiene after acceptance.

The radiological examination was performed using a CBCT scan (ProMax 3DS, Planmeca, Helsinki, Finland; voxel size of 0.075 mm), obtaining a series of DICOM images.

**Figure 1:**
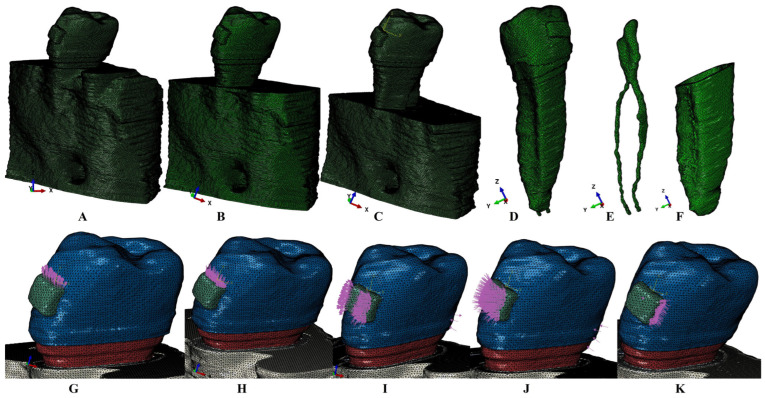
Mesh model of one of nine 3D models: (**A**) second lower right premolar model with 1 mm horizontal periodontal breakdown intact periodontium, (**B**) 4 mm bone loss model, (**C**) 8 mm bone loss model, (**D**) second lower premolar with the NVB and pulp, (**E**) the dental pulp and NVB of the second lower premolar, and (**F**) the PDL with 1 mm tissular loss. Applied vectors: (**G**) extrusion, (**H**) intrusion, (**I**) rotation, (**J**) tipping, and (**K**) translation.

The reconstruction software was AMIRA 5.4.0 (Visage Imaging Inc., Andover, MA, USA). Each dental tissular component was manually and individually identified, selected, and reconstructed. Thus, the enamel, dentine, dental pulp, periodontal ligament, cementum, cortical, and trabecular bone were reconstructed and assembled into a single 3D model (a model per patient) (Figure 1). The missing bone and periodontium were reconstructed (to obtain models with an intact periodontium), as well as the base of a stainless-steel bracket. The periodontal ligament had a variable thickness of 0.15–0.225 mm. The cementum was reconstructed as dentin because of their similar physical properties and difficulties in their separation (Table 1). The first molar and premolar alveolar sockets were filled with cortical and alveolar bone, guarding only the second premolar (Figure 1). A gradual horizontal periodontal breakdown was simulated by reducing each intact periodontium model by 1 mm, up to 8 mm of bone loss. The total number of 3D models thus obtained reached seventy-two.

The mesh models had 5.06–6.05 million C3D4 tetrahedral elements, 0.97–1.07 million nodes, and a global element size of 0.08–0.116 mm. No element errors were found, and only a few element warnings were found in the non-essential areas. The highest number of warnings for the pulp–NVB was four, about 0.0158% of the total 25,252 elements of one of the nine intact periodontium models. All internal algorithm checks were successfully passed.

The numerical analysis was performed in ABAQUS 6.13-1 (Dassault Systèmes Simulia Corp., Maastricht, The Netherlands), by employing the Tresca (non-homogenous, shear) and Von Mises (homogenous, overall) methods, suitable for ductile resemblance tissues [8,9,11,32,33,34,37]. Two orthodontic forces of 0.5 N/5 KPa and 4 N/40 KPa and five movements, extrusion, intrusion, translation, rotation, and tipping, were simulated (Figure 1). The boundary assumptions were non-homogeneity/homogeneity, linear elasticity, isotropy, and perfect bonded interfaces, as in recent numerical dental studies [4,5,6,7,38,39,40,41,42,43,44,45,46,47,48,49,50,51,52,53,54,55,57,58].

The ABAQUS results were a series of color-coded displays of the stress distribution of various intensities ranging from red—high to blue—low intensity. The quantitative values were compared with the local physiological maximum hydrostatic pressure of 16–22 KPa. Then, the exhibited tissular biomechanical behavior was compared with other numerical reports and known clinical data.

## 3. Results

**Figure 2:**
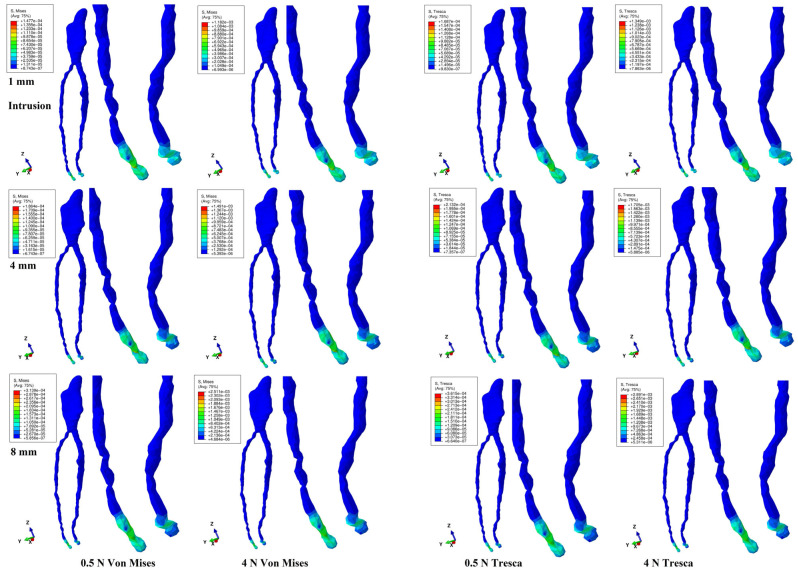
Intrusion movement for 1, 4, and 8 mm periodontal breakdown—comparative stress distribution in one of the nine 3D models using the Von Mises and Tresca numerical methods, for 0.5 and 4 N applied forces.

**Figure 3:**
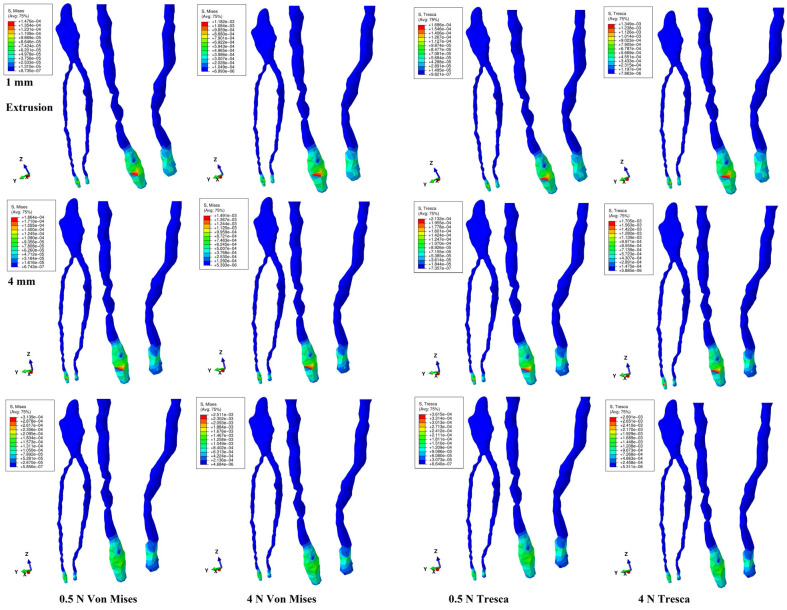
Extrusion movement for 1, 4, and 8 mm periodontal breakdown—comparative stress distribution in one of the nine 3D models using the Von Mises and Tresca numerical methods, for 0.5 and 4 N applied forces.

**Figure 4:**
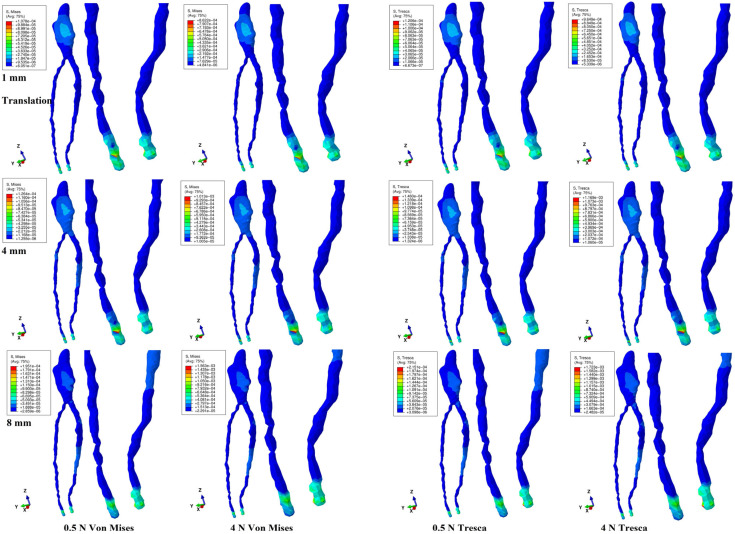
Translation movement for 1, 4, and 8 mm periodontal breakdown—comparative stress distribution in one of the nine 3D models using the Von Mises and Tresca numerical methods, for 0.5 and 4 N applied forces.

**Figure 5:**
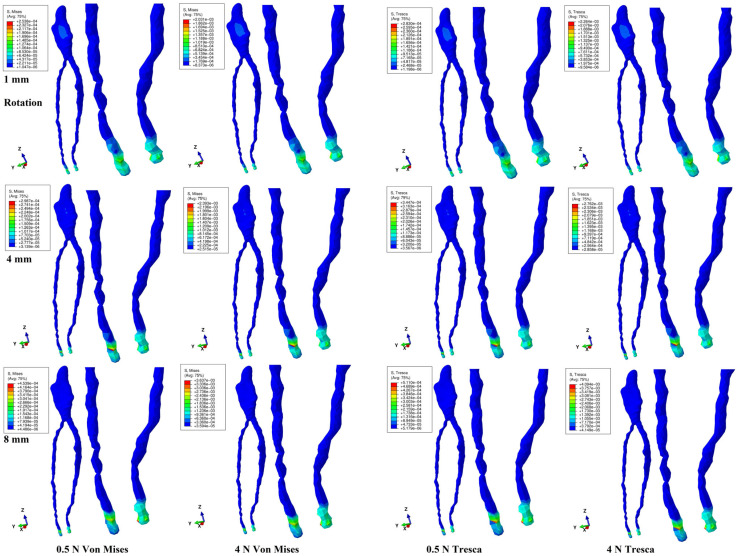
Rotation movement for 1, 4, and 8 mm periodontal breakdown—comparative stress distribution in one of the nine 3D models using the Von Mises and Tresca numerical methods, for 0.5 and 4 N applied forces.

**Figure 6:**
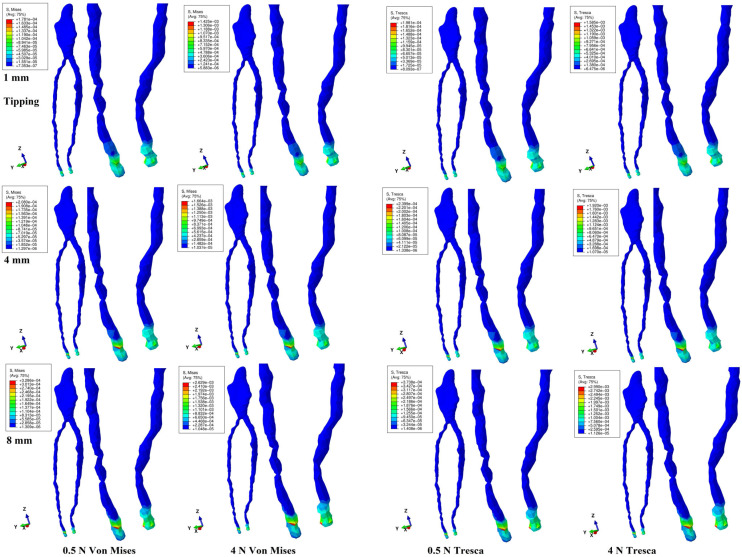
Tipping movement for 1, 4, and 8 mm periodontal breakdown—comparative stress distribution in one of the nine 3D models using the Von Mises and Tresca numerical methods, for 0.5 and 4 N applied forces.

Both methods showed that during a gradual horizontal periodontal breakdown process, only a small fraction of the applied orthodontic load reached the dental pulp and NVB (Figure 2, Figure 3, Figure 4, Figure 5 and Figure 6 and Table 2). The quantitative results displayed with the Tresca method were higher than those shown using Von Mises. Thus, the numerical difference was an average of 1.14 times (1.17 times for the NVB and 1.12 times for dental pulp), meeting the average scientific specified interval of 1.15 times (around 10%). During the entire periodontal breakdown simulation of 1–8 mm tissular loss, the quantitative results were lower than the local maximum physiological hydrostatic pressure of 16 KPa (Table 2). The NVB quantitative stress was 5.71 (1 mm loss) to 10.73 times (4 and 8 mm loss) higher than the dental pulp stress (Tresca method). The quantitative results showed a gradual stress increase for the NVB and dental pulp, strictly correlated with bone loss. The qualitative results displayed a similar color-coded stress distribution for both methods.

However, when assessing the two methods, it must be emphasized that Tresca was designed for non-homogenous ductility and Von Misses was designed for homogenous ductility, while dental tissues are considered non-homogenous ductile resemblance materials.

Both orthodontic forces displayed visible stress at the NVB level for five movements during the horizontal periodontal breakdown process, but almost none for dental pulp (except translation).

Intrusion (Figure 2) and extrusion (Figure 3) movements showed visible tissular NVB deformation (i.e., compression for intrusion and tension for extrusion) and no pulpal stress. During extrusion, limited red color-coded high-intensity areas at the NVB level were visible at up to 4 mm of bone loss, totally disappearing at 8 mm of loss (Figure 3). At 8 mm of loss, there was a doubling of the stress amount when compared with the 1 mm level (Table 2). From the initial 4 N/40 KPa of orthodontic loads, only 1.12 KPa (about 2.8% for 1 mm) to 2.88 KPa (7.2% for 8 mm) reached the NVB, while for the pulp, the average values were 0.11 KPa (0.275%, 1 mm) to 0.25 KPa (0.625%, 8 mm) using the Tresca method. Thus, during the two movements, about 92.8–97.2% of the initial applied orthodontic loads were absorbed and dissipated before arriving at the NVB level.

Translation (Figure 4) is the only movement to display visible pulpal involvement, both coronal and radicular. The coronal pulp displayed an extended blue-color-coded low-intensity stress area on the mesial, distal, and vestibular sides, with a higher extent at 1 mm of loss, progressively reducing at 4 mm and disappearing at 8 mm. The radicular pulp showed no involvement at 1 mm, progressively affecting the cervical and middle thirds at 4 mm and spreading to the apical third at 8 mm. The NVB displayed small red-color-coded high-intensity stress up to 4 mm loss, disappearing at 8 mm loss. These stress areas were similar for both forces and methods, indicating that the translation movement was the only one to induce pulpal stress. Quantitatively, the NVB stress amount was the smallest among the five movements. From the initial 4 N/40 KPa of orthodontic loads, only 0.85 KPa (about 2.12% for 1 mm) to 1.72 KPa (4.3% for 8 mm) reached the NVB, while for the pulp, the average values ranged from 0.15 KPa (0.375%, 1 mm) to 0.24 KPa (0.6%, 8 mm), using the Tresca method. Thus, during translation, about 95.7–97.88% of the initial applied orthodontic loads were absorbed and dissipated before arriving at the NVB level.

Rotation (Figure 5) displayed small low-intensity stress on the mesial and distal sides of the coronal pulp at 1 mm loss, disappearing at 4 mm. No other visible pulpal stress was displayed. The NVB displayed small red high-intensity color-coded areas after 4 mm of loss. Quantitatively, rotation displayed the highest stress for the NVB and pulp among the five movements. From the initial 4 N/40 KPa of orthodontic loads, only 1.2 KPa (about 3% for 1 mm) to 4.08 KPa (10.2% for 8 mm) reached the NVB, while for the pulp, the average values were 0.21 KPa (0.525%, 1 mm) to 0.38 KPa (0.95%, 8 mm), using the Tresca method. Thus, during rotation, about 89.8–97% of the initial applied orthodontic loads were absorbed and dissipated before arriving at the NVB level.

Tipping (Figure 6) displayed NVB stress, with limited high-intensity red-color-coded stress areas for 4–8 mm loss. Quantitatively the tipping movement displayed the second highest stress amounts after rotation for the NVB and pulp. From the initial 4 N/40 KPa of orthodontic loads, only 1.21 KPa (about 3% for 1 mm) to 2.98 KPa (7.45% for 8 mm) reached the NVB, while for the pulp, the average values were 0.17 KPa (0.425%, 1 mm) to 0.26 KPa (0.65%, 8 mm), using the Tresca method. Thus, during tipping, about 92.55–97% of the initial applied orthodontic loads were absorbed and dissipated before arriving at the NVB level.

Biomechanically, during the periodontal breakdown, the applied orthodontic forces displayed visible effects, especially at the NVB level, in terms of both tissular deformations (intrusion and extrusion), as well as limited red high-intensity color-coded stress areas (extrusion, translation, rotation, and tipping). Quantitively, during the periodontal loss and under 4 N of applied force, all amounts of stress were lower than the MHP; thus, in intact healthy tissues, the local ischemic risks were evaluated as low. However, the above-mentioned biomechanical behavior could induce ischemic risks if the tissues were previously injured (a NVB subjected to occlusal trauma, dental pulp with direct–indirect pulpal capping), compromising the tissue’s natural ability to sustain damage.

From the initial 4 N/40 KPa of applied orthodontic loads, only around 1–4 KPa manifested as stress at the NVB level. Both methods have similarly shown rotation to quantitatively be the most stressful movement for the NVB and pulp, closely followed by tipping, extrusion, and intrusion. Thus, for 1 mm bone loss, only 2–3% of the applied force manifests at the NVB level (0.27–0.5% for pulp), while for 8 mm loss, the received stress is 4–10% for the NVB (0.6–0.9% for pulp) when compared to the applied one. The tissular absorption–dissipation ability of the tooth (enamel, dentine, and stainless-steel bracket) is around 97% for 1 mm bone loss and around 90–93% for 8 mm loss. An even higher absorption–dissipation rate of the tooth (i.e., enamel, dentine, and bracket) of around 99% is seen when quantitatively assessing the pulpal stress during periodontal breakdown. The above-observed quantitative difference between the NVB and pulp is due to the anatomical topography of the two structures and different biomechanical behavior. Thus, the pulp is contained and isolated in a hard-wall pulpal chamber and root canals while the NVB is held in the PDL apical third with an absorption–dissipation role.

The 0.5 N/5 KPa applied force displayed similar qualitative results with the 4 N load, while the quantitative results were eight times smaller.

## 4. Discussion

Our study included 1440 numerical simulations over 72 3D models of the second lower premolar. It aimed to assess how much of orthodontic force applied at the bracket level during a 1–8 mm gradual horizontal periodontal breakdown would affect the NVB and dental pulp during five orthodontic movements. It must be emphasized that this is the first study to approach these issues (except our previous work [8,9,11,32,33,34]).

Only a few numerical studies are available investigating the biomechanical behavior of pulp and the NVB, and most belong to our team. Numerical studies are the only approach to individually studying these small tissues. Our study is the first to investigate absorption–dissipation issues during periodontal breakdown, providing data for both the clinician and the researcher. When planning and assessing the outcome of orthodontic treatment, especially in cases with periodontal breakdown, knowing the absorption–dissipation ability of dental tissues and how much applied orthodontic load reaches the dental pulp and NVB is important.

We used only two numerical methods suitable for dental tissues, respectively, Tresca (for non-homogenous materials) and Von Mises (for homogenous materials), specially designed for ductile resemblance materials [8,9,11,32,33,34]. Dental tissues are considered non-homogenous ductile resemblance materials with a certain brittle flow mode [8,9,11,32,33,34,37]; thus, Tresca is quantitatively better suited. Two orthodontic forces were used (a light 0.5 N force and a moderate 4 N force) to see if there were differences in the tissue’s biomechanical behavior. Moreover, since numerical analysis accuracy is significantly influenced [8,32,33,34,37] by boundary assumptions (homogeneity/non-homogeneity, linear elasticity/non-linear elasticity, and isotropy/anisotropy), it is important to address all the points mentioned above [4,5,6,7,15,16,17,18,35,38,39,40,41,42,43,44,45,46,47,48,49,50,51].

Qualitatively, both numerical methods displayed a similar color-coded stress display and tissular deformations. Quantitatively, Tresca was 1.14 times higher than VM, in line with the scientific interval of 1.15 times and previous reports [8,9,11,32,33,34].

The 1–8 mm bone loss simulation showed that only a small percentage of the initially applied orthodontic force produced effects at the pulp and NVB levels. Thus, for 1 mm bone loss, only 2–3% of the applied force manifests at the NVB level (0.27–0.5% for pulp), while for 8 mm loss, the received stress is 4–10% for the NVB (0.6–0.9% for pulp) when compared to the applied one. When assessing the NVB, more than 90% of the stress (97% for 1 mm loss and 90–93% for 8 mm loss) is absorbed and dissipated by the enamel, dentine, stainless steel brackets, and PDL. An even higher absorption–dissipation of 99% is seen for dental pulp. The main difference between the two tissues is due to their anatomical topography and biomechanical behavior: dental pulp is contained in the hard walls of the incompressible pulp chamber and root canals, while the highly deformable PDL apical third holds the NVB. These findings are in line with our previous reports [8,9,11]. Our previous study [11] reported that 97.2–99.9% of loads are absorbed and dissipated by the enamel, dentine, and stainless steel brackets in the intact periodontium, while only 2.8% of the applied load induces stresses in the NVB and 0.02–0.5% in the dental pulp. By correlating the two studies, the periodontal breakdown biomechanically reduces tooth tissue absorption–dissipation. In an earlier study [8], the individual tissular absorption–dissipation ability of dentine (40–93%), enamel (40–65%), and stainless-steel brackets (16%) were reported for various levels of reduced periodontium. The same report [8] revealed the percentage of stress that affected the PDL (0.3–8.4%), NVB (0.2–0.7%), and pulp (0.02–0.7%). Another numerical analysis [9] revealed that dental tissue holds an absorption–dissipation ability of 85% (with stresses being dissipated before reaching the circulatory sensitive tissues: 86.66–97.5% for the PDL, 98% for the NVB, and 99.6–99.94% for pulp). All three reports are in line with the results reported herein, signaling the biomechanical influence of bone loss over the tissue’s stress absorption–dissipation ability.

Moreover, the quantitative results both herein and in previous reports [8,9,11] were lower than 16–22 KPa (the local physiological maximum hydrostatic pressure), seeming not to induce any ischemic risks for intact and healthy NVBs and dental pulp (due to the natural tissular ability to sustain damage) [1,2,3,15,18,24,27,28,29,30] during orthodontic movements. These findings are in line with that of other reports [4,5,6,7,47,48,49,50,51]. However, if there is previous occlusal trauma (for NVB) [19,20] and/or dental pulp injuries (direct and indirect pulp capping) [3,22,23,25,26,31], due to morpho-pathological tissular changes [59], this natural ability to adapt and sustain damage is diminished (internal histo-morpho-pathological changes) [14,28,29,31,36] and ischemic risks could appear [11] during the orthodontic treatment. Thus, if a light and/or moderate orthodontic force [10] induces no ischemic risks during movements in the intact healthy periodontium, a similarly applied force in the reduced periodontium and the presence of occlusal trauma and/or injured pulp could be prone to significant ischemia [12,13,15,21,23,24,25,31] even if the local MHP is not exceeded. Thus, the translation movement could inflict the above-mentioned pulpal problems during orthodontic treatments for cases with periodontal loss and should be considered with care, especially if the tooth displays various signs of previous dental treatments. The tissular biomechanical behavior reported herein, the previous numerical report [11], and clinical data [3,19,20,22,23,25,26,31] back the findings mentioned above.

Biomechanically, the engineering field sees 0.5 and 4 N as tiny forces [8,9,11,32,33,34], while clinically, the tissular displacements and deformations are also small [10]. Thus, the boundary assumptions proposed by the engineering field (linear elasticity, isotropy, and homogeneity/non-homogeneity) are biomechanically and physically correct for dental numerical studies [4,5,6,7,15,16,17,18,35,38,39,40,41,42,43,44,45,46,47,48,49,50,51]. Nevertheless, the selected failure criteria are crucial in describing the tissular biomechanical behavior as brittle, ductile, and liquid [37]. The brittle physical–mechanical behavior implies that under a load, the material suffers tiny or no deformation (no recovery of the initial form), followed by necking, cracking, and destructive breaking. The ductile behavior implies that under a load, elastic deformation is displayed, with the recovery of the initial form when the force ceases. The liquid does not deform, does not show any shear stress, and has no compressive/tensile deformation. In the engineering field, each numerical method is designed to describe a certain behavior better. Thus, the brittle behavior is described by the maximum (tensile) and minimum (compressive) principal stress, the ductile behavior by Tresca (non-homogenous) and Von Mises (homogenous), while liquids are investigated using hydrostatic stress. Any misuse of the failure criteria will lead to a loss of accuracy and contradictory results, as proven by previous comparative analyses [8,9,11,32,33,34]. Moreover, many dental numerical studies disregarded this mandatory requirement with reports contradicting the clinical data [4,5,6,7,38,39,40,41,42,43,44,45,46,47,48,49,50,51,52,53,54]. Thus, Wu et al. used hydrostatic stress [4,5,47] to investigate the optimal orthodontic force in premolar rotation, reporting 2.1–2.9 N as optimal. However, the stress distribution showed only apical stress and no cervical stress, which is biomechanically incorrect. In another numerical analysis using the same hydrostatic stress, Hohman et al. [6,7] reported 80 KPa for 1 N of intrusion, and 40 KPa for 3–6 N of lingual torque, highly exceeding the 16–22 KPa of the MHP and implying extensive ischemia with necrosis that clinically does not appear. A similar issue is related to the linearity vs. non-linearity of periodontal ligaments, with studies reporting contradictory results [38,39,40] due to the use of the brittle-like method for ductile tissue. The same problem is still perpetuated by the current numerical studies using brittle and hydrostatic mathematical models when describing the ductile tissular biomechanical behavior [48,49,50,51,55]. Only a few studies employed the correct Von Mises criteria [56,57]. The maximum tensile and minimum compressive stress are specific to a brittle material that suffers minimum deformation and necking closely followed by cracking, which cannot accurately describe the elastic deformation of the PDL, dentine, or dental pulp [48,49,50,51,58] (the same with pressure criteria [55]). Qualitative results [4,5,6,7,38,39,40,41,42,43,44,45,46,47,48,49,50,51,52,53,54,55,57,58] can provide stress distributions, but they cannot show the correct biomechanical pattern and quantitatively will not be in line with clinical knowledge and the MHP [8,9,11,32,33,34]. Only one other older study (except ours) was found to argue these above-mentioned differences [37].

The anatomical accuracy of 3D numerical models is important for obtaining the correct data. Models with a larger number of nodes and elements and a smaller global element size provide more accurate results. Most of the dental numerical studies [4,5,6,7,15,16,17,18,35,38,39,40,41,42,43,44,45,46,47,48,49,50,51,55,56,57,58] employed simpler models that affected the stress distribution accuracy (e.g., 1674/5205–23,563/32,812–1.67 million elements/nodes and 1.2 mm element global size vs. the 5.06–6.05 million/0.97–1.07 million elements/nodes and 0.08–0.116 mm used herein). The sample size is important, since in numerical analyses, a sample size of one is considered proper due to the method’s versatility in simulating various situations. However, a larger sample size will increase the accuracy and validity of the results (nine herein vs. one in most studies [4,5,6,7,15,16,17,18,35,38,39,40,41,42,43,44,45,46,47,48,49,50,51,53,54,57,58]).

Regarding the tissular deformations and stress deformations displayed during the periodontal breakdown (Figure 1 and Figure 2), intrusion and extrusion were better seen in the NVB, in line with other studies’ findings regarding the stressfulness of intrusion [2,6,7]. Additionally, all movements and bone loss levels showed NVB-limited red high-intensity color-coded areas. The main difference between the stress distribution herein and previous studies [2,6,7] is related to stress distribution (the natural localized various-intensity stress ranging from red—high to blue—low intensity vs. the extended unnatural areas of red—high intensity), due to the misuse of the hydrostatic pressure method for ductile resemblance tissues.

Pulpal stress was displayed only with translation and rotation. Thus, during the entire periodontal breakdown, translation showed coronal pulp stress areas (lower on the vestibular side and higher on the mesial and distal sides) and radicular pulp, indicating its high stressfulness for the dental pulp, consistent with the previous report [11]. Despite the qualitative decrease in coronal stress correlated with bone loss (Figure 4), the radicular involvement extended, showing the influence of periodontal loss over the pulp and movement’s stressfulness. Similar biomechanical behavior was displayed by rotation (1–4 mm loss, Figure 5), with a decreasing pulpal coronal stress tendency on the proximal sides but with no radicular involvement, as previously reported [11]. Nevertheless, quantitatively, rotation displayed the highest amount of stress, lower than MHP (in line with [4,5,11,47]), while translation was the lowest (in line with [11]). Biomechanical behavior was consistent with previous clinical reports [3,19,20,22,23,25,26,31].

The limitations of numerical studies are related especially to the proper use of the above-mentioned mandatory conditions. The biological variability (e.g., age, pulp condition, and trauma) can also influence the outcomes of the orthodontic treatment but will not impact the biomechanical stress distribution displayed herein. The experimental validation of tissular biomechanical behavior cannot be performed in vivo. However, indirect validation through correlations can enhance conclusions.

Numerical studies are accurate if properly conducted, moving closer to the accuracy of clinical and animal studies as reported by Zhang et al. [52]. There are reports regarding the poor quality of in vivo dental studies [13,14,35], raising concerns regarding some data used for the direct/indirect validation of numerical studies. The numerical analysis herein showed that the NVB and pulp received only a small percentage of the applied orthodontic force due to the tissular absorption–dissipation ability of the dentine (most of the tooth structure), enamel, and stainless-steel bracket, confirming the relative safety of 4 N force during periodontal breakdown. Nevertheless, it also showed that due to tissular deformations and stress distributions, as well as unpredictable internal tissular degenerative changes [14,28,29,31,36], even if the stress amounts are lower than the local MHP, in periodontal breakdown conditions associated with previous occlusal trauma and/or pulpal injuries, the orthodontic movements should carefully consider all these factors and minimize the applied load [10]. This study showed high-force dissipation (around 90–99%) at the NVB and pulp level; nevertheless, the applied force must also be correlated with the periodontal ligament stress dissipation ability, and thus, the applied orthodontic force in cases of periodontal breakdown should be kept to a minimum. Further numerical simulations could improve the knowledge as well as the use of mini-screws and their biomechanical implications.

## 5. Conclusions

1. During the gradual horizontal periodontal breakdown simulation, both methods displayed a similar color-coded stress distribution in the NVB and pulp for the two orthodontic loads and five movements.
2. Quantitatively, for 0.5 and 4 N force, during the 1–8 mm bone loss simulation, the NVB stress was 5.7–10.7 times higher than the pulpal stress but lower than the local physiological maximum hydrostatic pressure, with no tissular ischemic risks.
3. A gradual tissular stress increase (doubling and tripling at 8 mm loss when compared with 1 mm) was seen, strictly correlated with the bone loss level for both forces and five movements.
4. For 1 mm bone loss, only 2–3% of the applied force manifested at the NVB level (0.27–0.5% for pulp), while for 8 mm loss, the received stress was 4–10% for the NVB (0.6–0.9% for pulp) when compared to the applied force.
5. When the NVB stress was assessed, the tissular absorption–dissipation ability of the tooth (i.e., enamel, dentine, and stainless-steel bracket) was around 97% for 1 mm bone loss and around 90–93% for 8 mm loss, with a similar pattern for both forces.
6. When the pulpal stress was assessed, an absorption–dissipation rate of around 99% in the tooth (i.e., enamel, dentine, and bracket) was seen for the 1–8 mm periodontal breakdown, similarly for both forces.
7. Only translation displayed pulpal stress (coronal and radicular) during the periodontal breakdown for both loads and methods, while all other movements displayed only NVB stress (most visible during intrusion and extrusion).
8. Despite the reduced amount of stress reaching the NVB and dental pulp, the orthodontic treatments for cases of periodontal loss should be considered with care, especially for the teeth displaying various signs of previous dental treatments and occlusal trauma.

## 6. Practical Implications

Only a few numerical studies are available investigating the biomechanical behavior of pulp and the NVB, and most belong to our team. Numerical studies are the only approach to individually studying these small tissues. Our study is the first to investigate the absorption–dissipation issues during periodontal breakdown, providing data for both the clinician and the researcher. When planning and assessing the outcome of the orthodontic treatment, especially in cases of periodontal breakdown, knowing the absorption–dissipation ability of dental tissues and how much applied orthodontic load reaches the dental pulp and NVB is important. Thus, 4 N of applied load does not induce any ischemic risks in the pulp and NVB for the five orthodontic movements and during the 1–8 mm periodontal breakdown. Among the five, translation is the only one that induces coronal stress, while all other movements induce only NVB stress, which is important when conceiving the orthodontic treatment plan in reduced periodontium cases. The visible tissular deformations in the NVB area and stress distribution are important if these tissues have been traumatized previously. This study, conducted in the reduced periodontium and healthy tissues, proved that both light and moderate orthodontic forces have similar quantitative tissular absorption–dissipation patterns and an absorption rate of 90–99%. Researchers could benefit from a better understanding of employing the finite element analysis in dental tissues, as well as a comparative description between the only two numerical methods that are suitable for dentistry.

## Figures and Tables

**Table 1:** Physical properties of materials.

Materials	Young’s Modulus, E (GPa)	Poisson Ratio, ʋ	Refs.
Enamel	80	0.33	[8,9,11,32,33,34]
Dentin/cementum	18.6	0.31	[8,9,11,32,33,34]
Pulp and NVB	0.0021	0.45	[8,9,11,32,33,34]
PDL	0.0667	0.49	[8,9,11,32,33,34]
Cortical bone	14.5	0.323	[8,9,11,32,33,34]
Trabecular bone	1.37	0.3	[8,9,11,32,33,34]
Stainless steel bracket (Cr-Co)	218	0.33	[8,9,11,32,33,34]

**Table 2:** Maximum stress average values (KPa) produced by 0.5 N and 4 N force in the NVB and coronal pulp.

Resorption (mm)			1	2	3	4	5	6	7	8
**Intrusion**	**Tresca**	NVB	**1.12**	**1.31**	**1.51**	**1.70**	**2.00**	**2.29**	**2.59**	**2.88**
**4 N/40 KPa**		c	**0.11**	**0.12**	**0.13**	**0.15**	**0.17**	**0.20**	**0.22**	**0.25**
	**VM**	NVB	**0.94**	**1.12**	**1.30**	**1.49**	**1.88**	**2.06**	**2.18**	**2.50**
		c	**0.10**	**0.11**	**0.12**	**0.13**	**0.15**	**0.17**	**0.19**	**0.21**
	**Tresca**	NVB	0.14	0.16	0.19	0.21	0.25	0.29	0.32	0.36
**0.5 N/5 KPa**		c	0.01	0.02	0.02	0.02	0.02	0.02	0.03	0.03
	**VM**	NVB	0.12	0.14	0.16	0.19	0.24	0.26	0.27	0.33
		c	0.01	0.01	0.01	0.02	0.02	0.02	0.02	0.03
**Extrusion**	**Tresca**	NVB	**1.12**	**1.31**	**1.51**	**1.70**	**2.00**	**2.29**	**2.59**	**2.88**
**4 N/40 KPa**		c	**0.11**	**0.12**	**0.13**	**0.15**	**0.17**	**0.20**	**0.22**	**0.25**
	**VM**	NVB	**0.94**	**1.12**	**1.30**	**1.49**	**1.74**	**2.06**	**2.18**	**2.50**
		c	**0.10**	**0.11**	**0.12**	**0.13**	**0.15**	**0.17**	**0.19**	**0.21**
	**Tresca**	NVB	0.14	0.16	0.19	0.21	0.25	0.29	0.32	0.36
**0.5 N/5 KPa**		c	0.01	0.02	0.02	0.02	0.02	0.02	0.03	0.03
	**VM**	NVB	0.12	0.14	0.16	0.19	0.22	0.26	0.27	0.31
		c	0.01	0.01	0.01	0.02	0.02	0.02	0.02	0.03
**Translation**	**Tresca**	NVB	**0.85**	**0.95**	**1.06**	**1.17**	**1.30**	**1.44**	**1.58**	**1.72**
**4 N/40 KPa**		c	**0.15**	**0.16**	**0.18**	**0.20**	**0.21**	**0.22**	**0.23**	**0.24**
	**VM**	NVB	**0.74**	**0.83**	**0.92**	**1.01**	**1.14**	**1.28**	**1.42**	**1.56**
		c	**0.18**	**0.18**	**0.19**	**0.20**	**0.20**	**0.20**	**0.21**	**0.21**
	**Tresca**	NVB	0.11	0.12	0.13	0.15	0.16	0.18	0.20	0.21
**0.5 N/5 KPa**		c	0.02	0.02	0.02	0.03	0.03	0.03	0.03	0.03
	**VM**	NVB	0.09	0.10	0.11	0.13	0.14	0.16	0.18	0.19
		c	0.02	0.02	0.02	0.02	0.03	0.03	0.03	0.03
**Rotation**	**Tresca**	NVB	**1.20**	**1.61**	**2.01**	**2.75**	**3.08**	**3.42**	**3.75**	**4.08**
**4 N/40 KPa**		c	**0.21**	**0.23**	**0.24**	**0.26**	**0.29**	**0.32**	**0.35**	**0.38**
	**VM**	NVB	**1.29**	**1.58**	**1.87**	**2.17**	**2.31**	**2.45**	**2.60**	**2.75**
		c	**0.19**	**0.20**	**0.21**	**0.22**	**0.26**	**0.29**	**0.33**	**0.36**
	**Tresca**	NVB	0.15	0.20	0.25	0.34	0.39	0.43	0.47	0.51
**0.5 N/5 KPa**		c	0.03	0.03	0.03	0.03	0.04	0.04	0.04	0.05
	**VM**	NVB	0.16	0.20	0.23	0.27	0.29	0.31	0.32	0.34
		c	0.02	0.02	0.03	0.03	0.03	0.04	0.04	0.04
**Tipping**	**Tresca**	NVB	**1.21**	**1.45**	**1.68**	**1.91**	**2.18**	**2.45**	**2.72**	**2.98**
**4 N/40 KPa**		c	**0.17**	**0.18**	**0.19**	**0.19**	**0.21**	**0.23**	**0.24**	**0.26**
	**VM**	NVB	**1.05**	**1.25**	**1.46**	**1.66**	**1.90**	**2.15**	**2.39**	**2.63**
		c	**0.15**	**0.15**	**0.15**	**0.15**	**0.17**	**0.19**	**0.21**	**0.23**
	**Tresca**	NVB	0.15	0.18	0.21	0.24	0.27	0.31	0.34	0.37
**0.5 N/5 KPa**		c	0.02	0.02	0.02	0.02	0.03	0.03	0.03	0.03
	**VM**	NVB	0.13	0.16	0.18	0.21	0.24	0.27	0.30	0.33
		c	0.02	0.02	0.02	0.02	0.02	0.02	0.03	0.03

NVB—neuro-vascular bundle, c—coronal pulp.

## Data Availability

Everything is in the manuscript.
